# Sleep Alterations in Female College Students with Migraines

**DOI:** 10.3390/ijerph17155456

**Published:** 2020-07-29

**Authors:** Daniel Rodríguez-Almagro, Alexander Achalandabaso-Ochoa, Esteban Obrero-Gaitán, María C. Osuna-Pérez, Alfonso Javier Ibáñez-Vera, Rafael Lomas-Vega

**Affiliations:** Department of Health Science, University of Jaén, Paraje Las Lagunillas s/n, 23071 Jaén, Spain; dralmagro4@gmail.com (D.R.-A.); eobrero@ujaen.es (E.O.-G.); mcosuna@ujaen.es (M.C.O.-P.); ajibanez@ujaen.es (A.J.I.-V.); rlomas@ujaen.es (R.L.-V.)

**Keywords:** migraine, sleep disorders, snoring, sleep disruptions, daytime somnolence

## Abstract

Background: Many factors are thought to potentially trigger migraines, among which sleep disturbances are one of the most frequently reported. Both sleep disorders and migraines affect more women than men. This study aims to analyze sleep alterations in young adult women with migraines and how they are related to the presence, frequency, intensity, and disability of migraines in this population. Methods: Fifty-one female university students with physician-diagnosed migraines and 55 healthy female university students completed surveys assessing demographic information and frequency, intensity, and disability of migraines and sleep quality variables. Results: No differences in sleep quality were found between migraine subjects and healthy women (*p* = 0.815), but women with migraines presented higher daytime somnolence (*p* = 0.010), greater sleep disruptions (*p* = 0.002), and decreased sleep adequacy (*p* = 0.019). The presence of a migraine was significantly related to daytime somnolence (*p* = 0.003) and sleep disruptions (*p* = 0.021). Migraine-related disability was associated with sleep disruptions (*p* = 0.002), snoring (*p* = 0.016), and a decreased quantity of sleep (*p* = 0.040). Migraine frequency was related to sleep disturbance (*p* = 0.003) and snoring (*p* < 0.001). The intensity of migraines was associated with sleep disruptions (*p* = 0.004). Conclusions: Our results suggest a relationship between migraines and sleep alterations.

## 1. Introduction

Sleep disorders are a major concern in maintaining health and they affect more women than men [1]. Several factors may influence sleep with stress being a major factor, which has been associated with insomnia, excessive daytime sleepiness, daytime dysfunction, and lack of rest due to sleep deprivation [2]. It has been suggested that both severe stress exposure and sleep disturbances might trigger the glial overactivation responsible for chronic pain processes [3].

Migraine is the sixth most prevalent disease worldwide, presenting a global prevalence of 14.4%, affecting particularly young adult to middle-aged women [4]. It generates an important individual impact [5] that, along with its high prevalence, leads to an elevated economic burden, which is estimated to have a total annual cost of 111,000 billion euros in Europe [6]. It has been suggested that both disturbances in the nociceptive descendent inhibitory pathways and sensitization processes of the trigeminal pathways are motives of the migraine chronification process [7,8].

Many factors are thought to potentially trigger migraines, among which sleep disturbances are one of the most frequently reported [9]. There is a vicious cycle linking sleep disorders and migraines [10]. Poor quality or poor duration of sleep could trigger a migraine attack [10]. Moreover, not only have a higher prevalence of insufficient sleep [11] and severe sleep disturbances been observed among migraine patients [12], but also a higher frequency of migraine attacks has been reported in those who present short sleep duration and poor sleep quality [13] due to sleep disorders such as snoring or sleep apnea [14]. Conversely, the coping behaviors of migraineurs (e.g., going to sleep early to relieve migraine attacks) can be factors precipitating and perpetuating sleep disturbances themselves [15]. The direction of the relationship between migraines and sleep disorders remains unknown; even so, sleep alterations as a chronic pain trigger [3] could play an important role in migraine development and chronicity.

The above led us to believe that young women with migraines have poorer sleep behaviors than young women without migraines and that sleep alterations may play an important role in migraine processes. The present study is aimed, firstly, at analyzing the quality of sleep in young adult women with migraines in comparison with healthy young women and, secondly, to analyze the presence of sleep alterations and how they are related to the presence, frequency, intensity, and disability of migraines in this population. Assessment of these relationships could allow for a better understanding of migraines, which may help clinicians to manage it more efficiently.

## 2. Materials and Methods

### 2.1. Study Design

A cross-sectional study was designed and implemented according to the guidelines for the communication of observational studies established in the Strengthening the reporting of observational studies in epidemiology (STROBE) guide [16]. This study was carried out in accordance with the Helsinki Declaration, good clinical practices, and all applicable laws and regulations, and was approved by the Ethics Committee of the University of Jaén (reference number ABR 7/17). Patients who met the eligibility criteria gave informed consent and completed questionnaires.

### 2.2. Participants

Contact with participants was established at the Health Sciences Faculty of the University of Jaén (Spain) through posters and digital announcements. All participants were young adult undergraduate and graduate women over 18 years of age. In April 2019, 123 women were screened to participate in the present study. After being duly informed about its specifics, 111 were enrolled, of which 106 subjects completed all questionnaires and evaluations. Data collection was carried out between April and June 2019 at the University of Jaén. To participate in the study, it was necessary to be female, a young adult undergraduate or graduate, and to sign the informed consent form. Exclusion criteria were the presence of cognitive disturbance, ocular disease, previous head or neck trauma, or any type of acquired brain damage (e.g., ischemic or hemorrhagic stroke or damage derived from intracranial intervention).

A physician (F.H.) classified the participants into the headache or non-headache categories following the criteria described in the third edition of the International Classification of Headache Disorders [17] at the time of inclusion.

**Figure 1:**
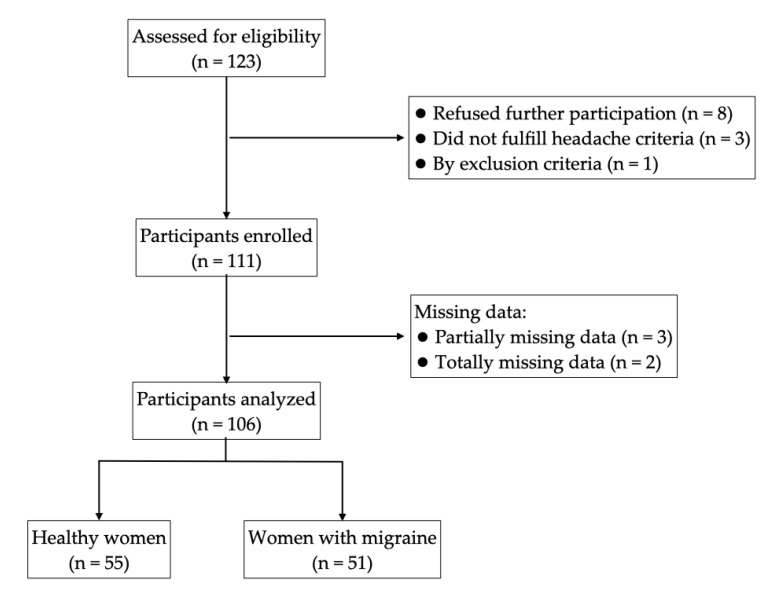
Flowchart of participant selection for the study.

The selection process of participants is graphically represented in Figure 1.

### 2.3. Measurements

Before completing the questionnaires, the participants reported their sociodemographic data including age, height, weight, and body mass index.

The Spanish version of the Medical Outcomes Study Sleep Scale (MOS-SS) was used to assess sleep quality [18]. The MOS-SS is a self-administered questionnaire comprised of 12 items in six different domains or sub-scales. From the MOS-SS questionnaire, the following variables were obtained: sleep disturbance (items 1, 3, 7, and 8; ICC = 0.78; 95% C.I. = 0.62–0.88); daytime somnolence (items 6, 9, and 11; ICC = 0.57; 95% C.I. = 0.30–0.75); sleep adequacy (items 4 and 12; ICC = 0.75; 95% C.I. = 0.56–0.87); snoring (item 10; ICC = 0.84; 95% C.I. = 0.71–0.91); sleep disruptions due to shortness of breath or with a headache (item 5; ICC = 0.84; 95% C.I. = 0.71–0.91); and quantity of sleep (item 2; ICC = 0.76; 95% C.I. = 0.58–0.87). In addition, the internal consistency of the MOS-SS questionnaire presented good Cronbach’s alpha values for all domains and indices ranging from 0.70 to 0.90. Due to our study purpose—to discriminate between good and poor sleepers—the Sleep Problems Index II that uses nine of the 12 items in the scale to compute an overall sleep problem summary (all items except 2, 10, and 11) MOS-SS questionnaire was employed. The Sleep Problems Index II presented a cut-off point of 33.33, with a sensitivity of 59.77% and a specificity of 91.18% [18].

The presence/absence of a migraine was determined through the medical diagnosis made at the beginning of the study. The degree of migraine-related disability as a way to estimate the personal impact of a migraine in terms of lost useful time as well as the frequency and intensity of a migraine, two of the most important criteria for a headache diagnosis, were estimated through the Spanish version of the Migraine Disability Assessment questionnaire [19]. Seven items compose this instrument: the first five items focus on three dimensions of daily life that can be affected by headaches while the other two items refer to the frequency and intensity of headaches. The sum of the score of the first five items provides the degree of headache-related disability while the sixth and seventh items indicate the frequency and intensity of migraine, respectively. This questionnaire presented excellent reliability values for the main scale (ICC = 0.81; 95% CI = 0.63–0.90; *p* < 0.001) and for headache frequency (ICC = 0.90; 95% CI = 0.79–0.95; *p* < 0.001), and good results for headache intensity (ICC = 0.63; 95% CI = 0.34–0.80; *p* < 0.001) as well as good internal consistency results (Cronbach’s alpha = 0.797) [19].

### 2.4. Sample Size Calculation

For the sample size calculation we required at least 10 observations per independent variable included in the multiple linear regression model [20], as well as at least 10 subjects per event in the multiple logistic regression model [21]. Given that the linear regression models employed a maximum of four variables (obtained in the bivariate analysis), for each predicted dependent variable related to a headache (i.e., presence, intensity, frequency, and disability), a minimum of 50 subjects in the migraine group were required for the purposes of our analysis.

### 2.5. Statistical Analysis

Management and statistical analyses were performed with the SPSS (Statistical Package for the Social Sciences) software Version 19.0 for Windows (SPSS Inc, Chicago, IL, USA). All analyses were performed with a 95% confidence level (α error = 0.05). The data were described using means and Standard Deviations (SD) for continuous variables and frequencies and percentages for categorical variables. To test the differences in sleep quality between migraine subjects and healthy women, the χ^2^ test was used.

First, a *t*-test was performed to test for differences in sleep alterations between young women with and without migraines where sleep disturbances, daytime somnolence, sleep adequacy, snoring, sleep disruptions due to shortness of breath or headache, and quantity of sleep were considered dependent variables and presence/absence of a migraine was considered as the independent variable. In this analysis, Cohen´s *d* was chosen as the effect size (ES) measure and calculated as the mean difference between the groups divided by the pooled SD of both groups. Cohen´s *d* can be interpreted as follows: ES less than 0.2 reflects a negligible effect; ≥0.2 and ≤0.5 reflects a small effect; ≥0.5 and ≤0.8 reflect a medium effect; and >0.8 reflects a large effect [22].

In order to analyze how sleep alterations may explain the presence/absence of migraine, the level of migraine-related disability, the intensity and frequency of migraines, and migraine-related variables were then considered as the dependent variables. Sleep disturbance, daytime somnolence, sleep adequacy, snoring, sleep disruptions due to shortness of breath or headache, and quantity of sleep were considered as independent variables.

The identification of the factors related to the presence/absence of a migraine was performed using a multiple logistic regression model due to the dichotomous nature of the dependent variable. The analysis included the entire sample and the relationships between the presence of migraines and the independent variables were analyzed using a simple logistic regression. The variables that presented a statistically significant odds ratio (*p* < 0.05) were selected for the multiple logistic model. A forward stepwise selection (conditional) method was used to add the independent variables to the multiple logistic model.

The prediction of continuous variables (intensity, frequency, and disability of headache) was performed using only data from the headache subjects. The relationships among each of the independent and dependent variables were explored by a simple linear regression. The dependent variables were intensity, frequency, and disability of headache. The factors related to the dependent variables were identified using a multiple linear regression model specific to each variable. The independent variables that presented a statistically significant β value (*p* < 0.05) were entered into each multiple linear model. A forward stepwise selection method was used to add the variables to the different multiple models.

The multivariate coefficient of determination (R^2^) was used to calculate the effect size in the linear regression models, whereas Nagelkerke’s R^2^ was used to calculate the effect size in the logistic regression model. According to Cohen, the R^2^ value can be considered insignificant when it is below 0.02, small when it is between 0.02 and 0.15, medium when it is between 0.15 and 0.35, and large when it is above 0.35 [22].

## 3. Results

In the present study 123 young women were initially assessed for participation, of which 111 were enrolled and 106 completed the planned measurements. Fifty-one young women were classified as migraine subjects and 55 were classified as healthy subjects. The results showed an elevated prevalence of poor sleepers among the study population (79.2%). Furthermore, 80.4% of women with migraines and 78.2% of healthy women were poor sleepers (Table 1). No differences in sleep quality were found between migraine subjects and healthy women (χ^2^ = 0.079; *p* = 0.815).

The analysis showed statistically significant differences in some sleep quality parameters between migraine subjects and healthy young women. Young women with migraines presented higher daytime somnolence, greater sleep disruptions, and decreased sleep adequacy. The between-group effect sizes in sleep quality parameters were medium (Table 2).

The multiple logistic model carried out to identify the factors related to the presence of migraines showed that the predictor variables were daytime somnolence and sleep disruptions due to shortness of breath or a headache (Table 3). The multiple model predicted 19% of the variance of the dependent variable (R^2^ = 0.190; *p* < 0.001).

The multiple linear regression model employed to identify the factors related to migraine-related disability showed that the predictor variables were sleep disruptions due to shortness of breath or headache, snoring, and quantity of sleep (Table 4). The multiple model predicted 32.3% of the variance of the dependent variable (R^2^ adjusted = 0.323; *p* = 0.040).

The multiple linear regression model used to identify the factors related to migraine frequency showed that the predictor variables were sleep disturbance and snoring (Table 5). The multiple linear model predicted 40.7% of the variance of the dependent variable (R^2^ adjusted = 0.407; *p* = 0.003).

The simple linear regression models performed to identify the bivariate relationships between migraine intensity and the predictor variables showed that only sleep disruptions were related to migraine intensity (Table 6), explaining the 13.9% of the variance of the dependent variable (R^2^ adjusted = 0.139; *p* = 0.004).

## 4. Discussion

The main findings of the present study were as follows. Both groups showed deficient sleep quality. Nevertheless, our findings underlined that the presence of migraines may be facilitated by bad sleep quality due to an increment in sleep disruptions and snoring, as well as decreased quantity of sleep. Furthermore, we observed a higher daytime somnolence, greater sleep disruptions, and decreased sleep adequacy among young women with migraines. In addition, we found that an increase in sleep disturbance was related to an increase in the intensity and frequency of migraines. Finally, we found an association between snoring and migraine frequency.

Poor sleep quality has been reported in students [23] and in post-menopausal women [24]. This is consistent with our results, in which a decrease in sleep quality was found in both groups. Furthermore, it was possible to observe an increase of daytime somnolence among young women with migraines as well as more sleep disruptive events and decreased sleep adequacy in comparison with young healthy women. Previously, it has been reported that patients with migraines not only presented less possibilities to report sleep adequacy [25], but also reported decreased daytime function due to sleepiness [26,27]. It has been suggested that sleep disturbances might cause aberrant glial activity which, in turn, increases the excitability of the neurons in the central nervous system through mechanisms such as long-term potentiation and increased synaptic efficiency thus allowing for chronicity in migraine subjects [3].

Item 5 of the MOS-SS questionnaire refers to sleep disruption as a cause of a headache or sleep apnea. Our results showed that sleep disruptions were related to the presence of migraines, to higher migraine intensity, and to an increase of migraine-related disability level. Due to our sample characteristics, we have interpreted that sleep disruptions were caused by headaches instead of sleep apnea since sleep apnea is less prevalent in women than in men [28] and obesity is the main risk factor for sleep apnea [28]. There is a consensus that sleep, especially during the deepest stages of sleep, is likely to play a restorative role in daytime activities [29]. It has been proposed that the therapeutic role of sleep in migraine attacks may be related to the restorative function that the glymphatic system has over the central nervous system [14]. Given that the glymphatic system is primarily active during sleep, it could be proposed that sleep disruption may lead to an accumulation of waste products, which may, in turn, contribute to the pathophysiology of migraines [14].

Moreover, we observed that snoring was associated with higher migraine frequency and greater migraine-related disability levels. Evidence has suggested that snoring should be considered in migraine processes [14]. Patients with chronic daily headaches are more likely to be daily snorers [30]. In this regard, snoring has been established as a significant risk factor for migraines [30,31], being strongly related to chronic migraines [32]. Intracranial and arterial pressure fluctuations during snoring may lead to individual susceptibility to pain progression, hypoxia, hypercapnia, and sleep fragmentation, which may be involved in migraine progression [33].

This study presents several limitations. First, we evaluated sleep parameters with self-reported questionnaires and an objective method (e.g., polysomnography) was not employed. Second, the study was conducted on a population from a very concrete geographical area and with a very specific age and any generalization of its results should be limited to individuals with similar characteristics. Therefore, our conclusions likely cannot be extrapolated to other population groups. Finally, the cross-sectional methodology used in this study does not allow causal relationships to be drawn.

In future studies, it will be interesting explore the possible sleep alterations in young women with migraines through an objective method such as polysomnography. In addition, exploring the influence of other structures involved in migraine development, such as cervical or vestibular disfunction, on sleep disorders could help clinicians in the management of sleep alterations.

## 5. Conclusions

In summary, a deficiency in sleep quality was observed among young women. Furthermore, young women with migraines showed an increase in sleep disruptions and snoring, as well as a decreased quantity of sleep in comparison with healthy young women. The presence of migraines was related to daytime somnolence and sleep disruptions. Migraine-related disability was associated with sleep disruptions, snoring, and a decreased quantity of sleep. Migraine frequency was related to sleep disturbance and snoring, while the intensity of the migraine was associated with sleep disruptions.

## Figures and Tables

**Table 1:** Description of the study participants.

		Total (*n* = 106)		Healthy Women (*n* = 55)		Migraine Women (*n* = 51)	
Categorical		F	%	F	%	F	%
Quality of sleep	Good sleepers	22	20.8	12	21.8	10	19.6
	Bad sleepers	84	79.2	43	78.2	41	80.4
**Continuous**		**Mean**	**SD**	**Mean**	**SD**	**Mean**	**SD**
Age (years)		21.25	21.25	21.24	3.54	21.25	2.73
Height (cm)		164.97	5.94	165.16	6.45	164.76	5.39
Weight (kg)		60.27	9.27	60.19	8.05	60.36	10.11
Midas		4.13	6.30	0.45	0.77	8.10	7.21
Headache frequency ^a^		4.56	8.21	2.39	2.50	7.00	11.09
Headache intensity (0/10) ^a^		4.83	2.36	3.85	2.18	5.88	2.09
Sleep disturbance ^b^		39.95	14.59	38.41	14.09	41.60	15.07
Daytime somnolence ^b^		42.77	13.66	39.50	12.64	46.30	13.95
Sleep adequacy ^b^		63.99	19.23	68.18	18.57	59.48	19.08
Snoring ^b^		23.27	12.74	23.33	13.83	23.20	11.58
Sleep disruptions ^b^		24.53	12.43	20.90	9.74	28.43	13.86
Quantity of sleep (hours) ^b^		7.05	1.04	7.15	1.11	6.931	0.95

^a^ Mean value of the last three months. ^b^ Variable obtained from the MOS-SS questionnaire. Abbreviations: MIDAS, Migraine Disability Assessment Questionnaire; F, Frequency; SD, Standard Deviation.

**Table 2:** Between-group differences in sleep parameters.

		Mean	SD	Mean Difference	95% C.I.		*t*	*p*	*d*
					Inferior	Superior			
Sleep disturbance ^a^	Healthy	38.42	14.10	3.18	−2.43	8.80	1.124	0.264	0.218
	Migraine	41.60	15.07						
Daytime somnolence ^a^	Healthy	39.49	12.64	6.80	1.68	11.92	2.634	0.010	0.513
	Migraine	46.30	13.95						
Sleep adequacy ^a^	Healthy	68.18	18.57	−8.70	−15.96	−1.45	−2.380	0.019	−0.462
	Migraine	59.48	19.08						
Snoring ^a^	Healthy	23.33	13.83	−0.131	−5.07	4.80	0–0.053	0.958	−0.008
	Migraine	23.20	11.58						
Sleep disruptions ^a^	Healthy	20.91	9.74	7.52	2.87	12.18	3.210	0.002	0.599
	Migraine	28.43	13.86						
Quantity of sleep ^a^	Healthy	7.16	1.11	−0.22	−0.62	0.18	−1.107	0.271	−0.228
	Migraine	6.93	0.95						

^a^ Variable obtained from the MOS-SS questionnaire. Abbreviations: SD, Standard Deviation; 95% C.I., 95% Confidence Interval.

**Table 3:** Univariate and multivariate logistic regression to analyze the factors related to the presence of migraines.

	Univariate Analysis				Multivariate Analysis			
Variable	OR	95% C.I.		*p*	OR	95% C.I.		*p*
		Inferior	Superior			Inferior	Superior	
Sleep disturbance ^a^	1.015	0.989	1.043	0.262	NS	NS	NS	NS
Daytime somnolence ^a^	1.040	1.008	1.073	0.013	1.057	1.006	1.073	0.003
Sleep adequacy ^a^	0.976	0.955	0.996	0.022	NS	NS	NS	NS
Snoring ^a^	0.999	0.970	1.030	0.958	NS	NS	NS	NS
Sleep disruptions ^a^	1.057	1.019	1.098	0.003	1.039	1.017	1.098	0.021
Quantity of sleep ^a^	0.808	0.551	1.183	0.273	NS	NS	NS	NS

^a^ Variable obtained from the MOS-SS questionnaire. Abbreviations: OR, Odds Ratio; 95% C.I., 95% Confidence Interval; NS, Non-Significant.

**Table 4:** Univariate and multivariate linear regression to analyze the factors related to migraine-related disability.

	Univariate Analysis				Multivariate Analysis			
Variable	β	95% C.I.		*p*	β	95% C.I.		*p*
		Inferior	Superior			Inferior	Superior	
Sleep disturbance ^a^	0.119	−0.014	0.252	0.078	NS	NS	NS	NS
Daytime somnolence ^a^	0.028	−0.120	−0.176	0.704	NS	NS	NS	NS
Sleep adequacy ^a^	−0.137	−0.238	−0.036	0.009	NS	NS	NS	NS
Snoring ^a^	0.224	0.057	0.390	0.010	0.205	0.80	0.329	0.002
Sleep disruptions ^a^	0.245	0.114	0.377	<0.001	0.184	0.036	0.332	0.016
Quantity of sleep ^a^	−2.203	−4.291	−0.115	0.039	−1.885	−3.676	−0.094	0.040

^a^ Variable obtained from the MOS-SS questionnaire. Abbreviations: B, Beta value; 95% C.I., 95% Confidence Interval; NS, Non-Significant.

**Table 5:** Univariate and multivariate linear regression to analyze the factors related to frequency of migraine.

	Univariate Analysis				Multivariate Analysis			
Variable	β	95% C.I.		*p*	β	95% C.I.		*p*
		Inferior	Superior			Inferior	Superior	
Sleep disturbance ^a^	0.311	0.120	0.503	0.002	0.257	0.094	0.420	0.003
Daytime somnolence ^a^	−0.147	−0.077	0.372	0.193	NS	NS	NS	NS
Sleep adequacy ^a^	−0.218	−0.373	−0.064	0.007	NS	NS	NS	NS
Snoring ^a^	0.534	0.306	0.762	<0.001	0.485	0.273	0.697	<0.001
Sleep disruptions ^a^	0.194	−0.029	0.417	0.086	NS	NS	NS	NS
Quantity of sleep ^a^	−2.622	−5.892	0.649	0.114	NS	NS	NS	NS

^a^ Variable obtained from the MOS-SS questionnaire. Abbreviations: B, Beta value; 95% C.I., 95% Confidence Interval; NS, Non-Significant.

**Table 6:** Univariate linear regression to analyze the factors related to the intensity of migraine.

	Univariate Analysis			
Variable	β	95% C.I.		*p*
		Inferior	Superior	
Sleep disturbance ^a^	0.035	−0.004	0.073	0.076
Daytime somnolence ^a^	−0.005	−0.048	0.038	0.811
Sleep adequacy ^a^	−0.010	−0.041	0.022	0.536
Snoring ^a^	−0.009	−0.061	0.043	0.726
Sleep disruptions ^a^	−0.059	0.020	0.099	0.004
Quantity of sleep ^a^	−0.431	−1.050	0.187	0.167

^a^ Variable obtained from the MOS-SS questionnaire. Abbreviations: B, Beta value; 95% C.I., 95% Confidence Interval.
